# Predictors of Clinical Success in the Treatment of Patients with Methicillin-Resistant *Staphylococcus aureus* (MRSA) Nosocomial Pneumonia (NP)

**DOI:** 10.1371/journal.pone.0131932

**Published:** 2015-07-21

**Authors:** Andrew F. Shorr, Laura A. Puzniak, Pinaki Biswas, Michael S. Niederman

**Affiliations:** 1 Pulmonary Critical Care; Washington Hospital Center, Washington, DC, United States of America; 2 Infectious Diseases and Vaccines; Pfizer Inc, Collegeville, Pennsylvania, United States of America; 3 Biostatistics, Pfizer Inc, Collegeville, Pennsylvania, United States of America; 4 Pulmonary and Critical Care Medicine, Weill Cornell Medical College, New York, New York, United States of America; University Hospital Münster, GERMANY

## Abstract

Methicillin-resistant *Staphylococcus aureus* (MRSA) remains an important pathogen in nosocomial pneumonia and is associated with significant morbidity and mortality. Clinical outcomes for nosocomial pneumonia are dependent on patient age, co-morbidities, severity of illness and appropriate antibiotic therapy. The objective of this secondary analysis was to identify baseline clinical variables that are associated with clinical success at the end of the study observation period. Data from a randomized blinded trial (NCT00084266) comparing linezolid (600-mg twice daily) to vancomycin (15-mg/kg twice daily, dose-adjusted) for the treatment of culture-proven MRSA pneumonia were analyzed to evaluate baseline clinical and demographic factors that may predict clinical success at end of study (EOS) (7-30 days after end of treatment). A multivariate logistic regression was conducted to identify baseline factors that are associated with clinical success. Patients treated with linezolid (OR 1.55 95% CI: 1.013, 2.355), no vasopressor receipt (OR 2.30, 95% CI: 1.303, 4.069), unilateral involvement (OR 1.70, 95% CI: 1.078, 2.681) and normal renal function (eGFR 30-80 vs >80 OR 0.48, 95% CI: 0.303, 0.750) were more likely to have clinical success. From a clinical standpoint, identifying reliable predictors of outcome and who might benefit more from one therapy versus another can help inform treatment decisions.

## Introduction

Methicillin-resistant *Staphylococcus aureus* (MRSA) remains an important pathogen in nosocomial pneumonia and is associated with significant morbidity and mortality. [1] Multiple factors correlate with outcomes in MRSA pneumonia such as patient age, co-morbidities, severity of illness and appropriate antibiotic therapy. [2–4]

As of May 2014, only two agents are approved by the US FDA for the treatment of MRSA nosocomial pneumonia: linezolid and vancomycin. In a recent double-blind randomized control trial (ZEPHYR) comparing linezolid to weight-based dosing of vancomycin, randomization to linezolid was associated with improved cure rates (57.6% vs. 46.6%)[5], although there was no difference in the 60-day mortality between treatment groups. This study is published [5] and the limitations including potential reasons for lack of difference in 60-day mortality between linezolid and vancomycin are discussed in detail in the original publication [5]. Although the baseline characteristics of the two treatment groups were similar[5], it remains important to understand how baseline patient characteristics interact with and affect cure rates. Additional explorations of potential relationships between baseline factors and eventual cure rates may also help clinicians identify subgroups of patients most likely to benefit from one of the two treatment options.

To explore this issue of baseline characteristics and outcomes, we conducted a secondary analysis of a randomized, blinded trial comparing linezolid to dose adjusted vancomycin for the treatment of MRSA. Specifically, the objective of this secondary analysis was to identify baseline clinical variables that are associated with clinical success at the end of the study observation period.

## Methods

### Patients

Data from a randomized blinded trial (NCT00084266) comparing linezolid (600-mg twice daily) to vancomycin (15-mg/kg twice daily, dose-adjusted) for the treatment of culture-proven MRSA pneumonia were analyzed to evaluate baseline clinical and demographic factors that may predict clinical success at end of study (EOS) (7–30 days after end of treatment). [5]. Patients from the mITT population (at least one dose of study treatment and a confirmed MRSA culture) with an observed clinical response at EOS were included in this secondary analysis. The key clinical outcome was categorized as “cure” (i.e., resolution of clinical pneumonia signs/symptoms vs. baseline, improved or no progression in all chest X-ray abnormalities, and no additional MRSA treatment required), “failure” (i.e., persistence or progression of baseline signs and symptoms of pneumonia after at least 2 days of treatment; progression of baseline radiographic abnormalities; development of new pulmonary or extrapulmonary clinical findings consistent with active infection), or “unknown” (i.e., extenuating circumstances precluded classification to the above) The complete trial report and methods have been previously reported. [5]

### Statistical analyses

The treatment-clinical response relationship was assessed to explore interactions between baseline variables and treatment. Stratified relative risk values and corresponding 95% confidence intervals (CIs) were calculated for each stratum-specific analysis.

Additionally, we conducted multivariate logistic regression to identify baseline factors that are associated with clinical success at the end of the study. [6] To reduce multicollinearity, a correlation analysis was conducted among clinically relevant baseline factors. Only the factors having the least average correlation were selected for inclusion into the full model. A final, reduced model was constructed from the full model via backward elimination with stay criteria (*α*) of 0.10. Average inclusion frequencies of each factor in the reduced model were calculated based on 1000 bootstrap samples to evaluate consistency of backward elimination [7]. The final, reduced model was then fitted with these factors and summarized using odds ratios (ORs) and 95% CIs. The Hosmer-Lemeshow test was used to measure goodness of fit. Statistical analyses were performed using the SAS version 9.2 (Cary, NC) software.

### Ethics statements

Written informed consent was obtained from all patients or their legally authorized representative. The study was approved by an institutional review board or ethics committee at each investigational center.

## Results

**Fig 1:**
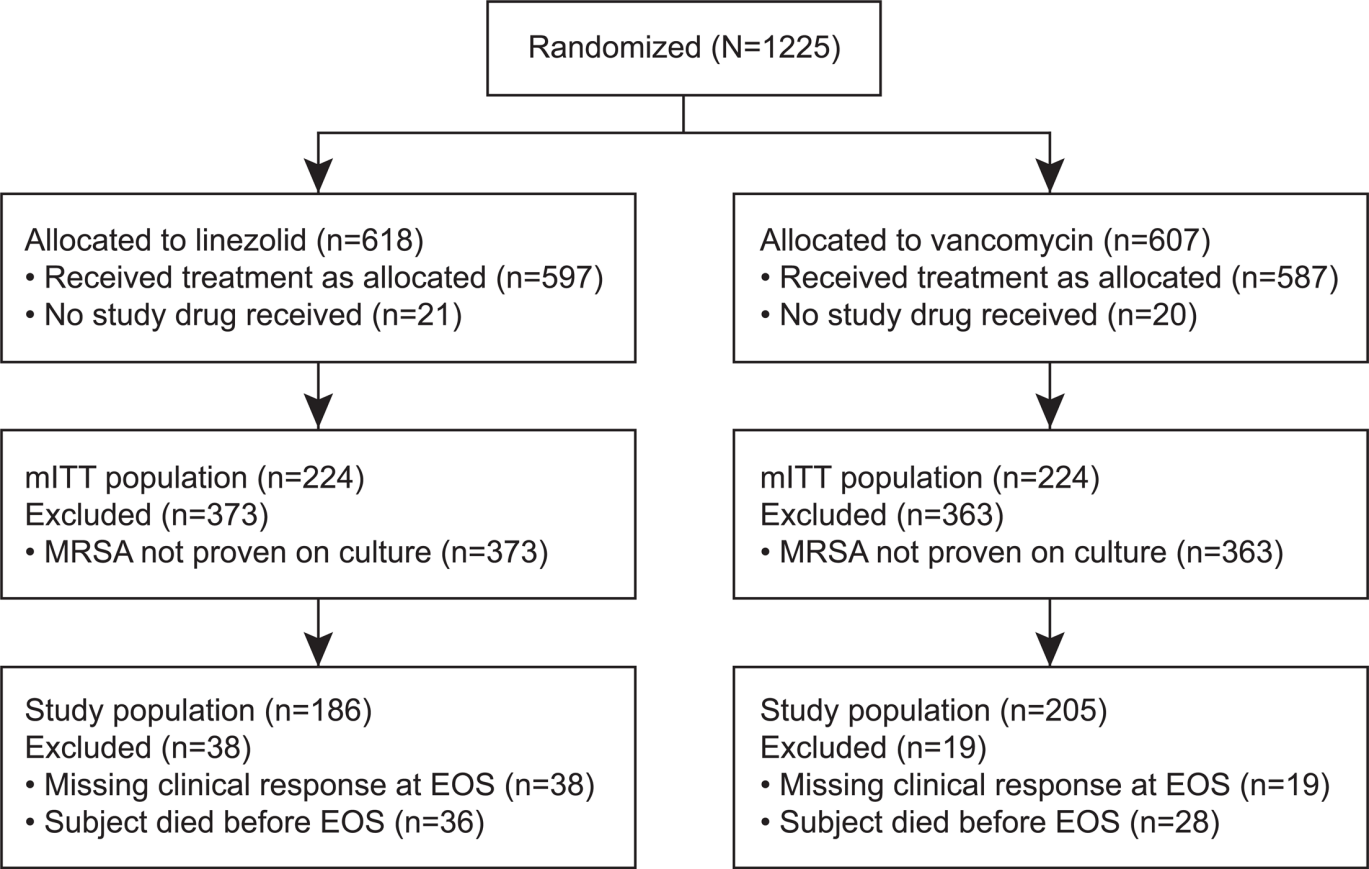
Flowchart showing study population.

There were 448 patients (224 treated with linezolid, 224 treated with vancomycin) in the mITT population (shown in Fig 1). Of these mITT patients, 186/224 (83%) linezolid treated patients and 205/224 (92%) vancomycin treated patients had a clinical response documented at EOS and were included in this analysis. There were no significant differences regarding the distribution of baseline characteristics between those treated with linezolid compared to vancomycin (shown in Table 1).

**Table 1:** Baseline characteristics by treatment regimen for analysis population (meeting mITT criteria and clinical response at EOS).

	Linezolid N = 186	Vancomycin N = 205	P value
Age, mean (SD)	61.8 (17.8)	61.4 (18.3)	0.82
Male gender, n (%)	124 (66.7)	130 (63.4)	0.53
Race, n (%)			0.80
White	130 (69.9)	136 (66.3)	
Black	21 (11.3)	30 (14.6)	
Asian	28 (15.1)	31 (15.1)	
Other	7 (3.8)	8 (3.9)	
Weight, n, mean (SD)	N = 186 78.0 (23.2)	N = 204 77.0 (21.1)	0.63
LTCF resident at baseline, n (%)	9 (4.8)	10 (4.9)	1.00
Type of subject, n (%)			0.23
Medical	106 (57.0)	111 (54.1)	
Surgical	49 (26.3)	46 (22.4)	
Trauma	31 (16.7)	48 (23.4)	
ICU at baseline, n (%)	156 (83.9)	181 (88.3)	0.24
Mechanical ventilation at baseline, n (%)	126 (67.7)	152 (74.1)	0.18
APACHE II score, n (%)			0.97
≤20	124 (66.7)	139 (67.8)	
>20	58 (31.2)	62 (30.2)	
Missing	4 (2.2)	4 (2.0)	
APACHE II score, mean (SD)	17.4 (6.3)	17.7 (6.1)	0.69
APACHE II score, median (IQR)	17.5 (10.0)	18.0 (9.0)	0.71
ACE/ARBs baseline, n (%)	24 (12.9)	30 (14.6)	0.66
Diuretics at baseline, n (%)	54 (29.0)	56 (27.3)	0.74
Pressors at baseline, n (%)	29 (15.6)	40 (19.5)	0.35
Corticosteroids at baseline, n (%)	43 (23.1)	44 (21.5)	0.72
Comorbidities n (%)			
Diabetes	66 (35.5)	89 (43.4)	0.12
Cardiac	108 (58.1)	123 (60.0)	0.76
Pulmonary	123 (66.1)	140 (68.3)	0.67
Renal/urinary	69 (37.1)	84 (41.0)	0.47
Gastrointestinal	109 (58.6)	108 (52.7)	0.26
Pathogen other than MRSA	84 (45.2)	91 (44.4)	0.92
eGFR (by MDRD mL/min/1.73m^2^)			0.66
MDRD <10–30	14 (7.6)	20 (9.8)	
MDRD 30 –<80	64 (34.4)	71 (34.6)	
MDRD ≥80	105 (56.5)	108 (52.7)	
Missing	3 (1.6)	6 (2.9)	
Chest x-ray			0.16
unilateral	51 (27.4)	72 (35.1)	
bilateral	134 (72.0)	133 (64.9)	
Missing	1 (0.5)	0	
Pleural effusion, n (%)	84 (45.2)	88 (42.9)	0.68
Bacteremia*, n (%)	21 (11.3)	35 (17.1)	0.11
Smoking status, n (%)			0.83
Current smoker	41 (22.0)	52 (25.4)	
Ex-smoker	86 (46.2)	86 (42.0)	
Non-smoker	58 (31.2)	66 (32.2)	
Missing	1 (0.5)	1 (0.5)	

ACE, angiotensin-converting-enzyme inhibitor; APACHE, Acute Physiology and Chronic Health Evaluation; ARB, angiotensin receptor blocker; eGFR, estimate glomerular filtration rate; ICU, intensive care unit; LTCF, long-term care facility; MDRD, Modification of Diet in Renal Disease

*Percentages may not add up to 100 because of rounding*.

* Within the full mITT population, there were 28/224 (12.5%) in the linezolid group and 38/224 (17.0%) in the vancomycin group with bacteremia.

The stratified analyses for clinical success at EOS are shown in Table 2. Baseline characteristics did not modify the association between treatment and clinical success. However, multiple subpopulations had significantly greater clinical success rates at EOS when treated with linezolid. Specifically females, the less severely ill (APACHE II score < 20 and not needing vasopressors), persons with diabetes, those not taking ACE/ARBs, and ex-smokers had higher EOS success rates among those treated with linezolid compared to vancomycin. There were no patient or baseline characteristics that had significantly greater clinical success rates with vancomycin compared to linezolid. In addition, key factors that have been previously associated with pneumonia outcome such as bacteremia at baseline, mechanical ventilation at baseline, and pleural effusion had similar clinical success rates regardless of whether these conditions were present or absent. For example, the relative risk for clinical success among those with bacteremia was 1.25 (95%CI: 0.64, 2.45) and those without bacteremia was 1.20 (95%CI: 0.97, 1.48). Furthermore, these three factors did not influence the final outcome and were not selected as significant predictors of clinical success in the multivariate or bootstrapping analysis.

**Table 2:** Stratified analyses for clinical success at EOS by treatment and clinical characteristics for analysis population (meeting mITT criteria and clinical response at EOS).

Parameter	Linezolid n/N (%)	Vancomycin n/N (%)	Risk Ratio(95% CI), p-value
**Overall (mITT)**	102/186 (54.8)	92/205 (44.9)	1.22 (1.00, 1.49), p = 0.055
**Age, y**			
≤65	55/96 (57.3)	48/95 (50.5)	1.13 (0.87, 1.48), p = 0.385
>65	47/90 (52.2)	44/110 (40.0)	1.31 (0.96, 1.77), p = 0.089
**Gender**			
Female	39/62 (62.9)	32/75 (42.7)	1.47 (1.07, 2.04), p = 0.025
Male	63/124 (50.8)	60/130 (46.2)	1.10 (0.85, 1.42), p = 0.530
**Race**			
White	66/130 (50.8)	59/136 (43.4)	1.17 (0.91, 1.51), p = 0.269
Black	12/21 (57.1)	15/30 (50.0)	1.14 (0.68, 1.91), p = 0.777
Asian	20/28 (71.4)	16/31 (51.6)	1.38 (0.92, 2.09), p = 0.181
Other	4/7 (57.1)	2/8 (25.0)	2.29 (0.59, 8.91), p = 0.315
**LTCF resident at baseline**			
No	96/177 (54.2)	89/195 (45.6)	1.19 (0.97, 1.46), p = 0.119
Yes	6/9 (66.7)	3/10 (30.0)	2.22 (0.77, 6.37), p = 0.179
**Type of patient**			
Medical	60/106 (56.6)	48/111 (43.2)	1.31 (1.00, 1.72), p = 0.058
Surgical	23/49 (46.9)	22/46 (47.8)	0.98 (0.64, 1.50), p = 1.000
Trauma	19/31 (61.3)	22/48 (45.8)	1.34 (0.88, 2.03), p = 0.249
**ICU at baseline**			
No	20/30 (66.7)	9/24 (37.5)	1.78 (1.00, 3.16), p = 0.054
Yes	82/156 (52.6)	83/181 (45.9)	1.15 (0.92, 1.42), p = 0.231
**Mechanical ventilation at baseline**			
No	36/60 (60.0)	27/53 (50.9)	1.18 (0.84, 1.65), p = 0.350
Yes	66/126 (52.4)	65/152 (42.8)	1.22 (0.96, 1.57), p = 0.118
**APACHE II score**			
≤20	75/124 (60.5)	65/139 (46.8)	1.29 (1.03, 1.62), p = 0.035
>20	26/58 (44.8)	25/62 (40.3)	1.11 (0.73, 1.69), p = 0.712
Missing	1/4 (25.0)	2/4 (50.0)	0.50 (0.07, 3.55), p = 1.000
**ACE/ARBs baseline**			
No	87/162 (53.7)	74/175 (42.3)	1.27 (1.01, 1.59), p = 0.039
Yes	15/24 (62.5)	18/30 (60.0)	1.04 (0.68, 1.59), p = 1.000
**NSAIDs at baseline**			
No	99/182 (54.4)	89/202 (44.1)	1.23 (1.01, 1.51), p = 0.052
Yes	3/4 (75.0)	3/3 (100.0)	0.75 (0.43, 1.32), p = 1.000
**Vasopressors at baseline**			
No	95/157 (60.5)	77/165 (46.7)	1.30 (1.05, 1.59), p = 0.014
Yes	7/29 (24.1)	15/40 (37.5)	0.64 (0.30, 1.38), p = 0.300
**Corticosteroids at baseline**			
No	80/143 (55.9)	72/161 (44.7)	1.25 (1.00, 1.57), p = 0.066
Yes	22/43 (51.2)	20/44 (45.5)	1.13 (0.73, 1.74), p = 0.670
**Baseline Diuretics**			
No	72/132 (54.5)	68/149 (45.6)	1.20 (0.95, 1.51), p = 0.152
Yes	30/54 (55.6)	24/56 (42.9)	1.30 (0.88, 1.91), p = 0.252
**Diabetes**			
No	64/120 (53.3)	57/116 (49.1)	1.09 (0.85, 1.39), p = 0.602
Yes	38/66 (57.6)	35/89 (39.3)	1.46 (1.05, 2.04), p = 0.034
**Cardiac comorbidity**			
No	50/78 (64.1)	42/82 (51.2)	1.25 (0.96, 1.64), p = 0.112
Yes	52/108 (48.1)	50/123 (40.7)	1.18 (0.89, 1.58), p = 0.289
**Pulmonary comorbidity**			
No	35/63 (55.6)	32/65 (49.2)	1.13 (0.81, 1.57), p = 0.485
Yes	67/123 (54.5)	60/140 (42.9)	1.27 (0.99, 1.63), p = 0.065
**Oncologic comorbidity**			
No	93/167 (55.7)	85/183 (46.4)	1.20 (0.98, 1.47), p = 0.088
Yes	9/19 (47.4)	7/22 (31.8)	1.49 (0.69, 3.23), p = 0.352
**Hepatobiliary comorbidity**			
No	87/157 (55.4)	81/177 (45.8)	1.21 (0.98, 1.50), p = 0.081
Yes	15/29 (51.7)	11/28 (39.3)	1.32 (0.74, 2.35), p = 0.429
**Renal/urinary comorbidity**			
No	67/117 (57.3)	54/121 (44.6)	1.28 (1.00, 1.65). p = 0.053
Yes	35/69 (50.7)	38/84 (45.2)	1.12 (0.81, 1.56), p = 0.519
**Gastrointestinal comorbidity**			
No	41/77 (53.2)	43/97 (44.3)	1.20 (0.88, 1.63), p = 0.286
Yes	61/109 (56.0)	49/108 (45.4)	1.23 (0.95, 1.61), p = 0.136
**Baseline pathogens**			
MRSA only	60/102 (58.8)	55/114 (48.2)	1.22 (0.95, 1.57), p = 0.134
MRSA mixed	42/84 (50.0)	37/91 (40.7)	1.23 (0.89, 1.71), p = 0.228
**MDRD**			
<30	7/14 (50.0)	10/20 (50.0)	1.00 (0.51, 1.98), p = 1.000
30-<80	27/64 (42.2)	24/71 (33.8)	1.25 (0.81, 1.93), p = 0.375
≥80	67/105 (63.8)	56/108 (51.9)	1.23 (0.98, 1.55), p = 0.096
Missing	1/3 (33.3)	2/6 (33.3)	1.00 (0.14, 7.10), p = 1.000
**Chest X-ray involvement**			
unilateral	35/51 (68.6)	37/72 (51.4)	1.34 (1.00, 1.79), p = 0.065
bilateral	67/134 (50.0)	55/133 (41.4)	1.21 (0.93, 1.57), p = 0.177
Missing	0/1		
**Pleural effusion**			
No	53/102 (52.0)	50/117 (42.7)	1.22 (0.92, 1.61), p = 0.178
Yes	49/84 (58.3)	42/88 (47.7)	1.22 (0.92, 1.62), p = 0.173
**Bacteremia**			
No	93/165 (56.4)	80/170 (47.1)	1.20 (0.97, 1.48), p = 0.101
Yes	9/21 (42.9)	12/35 (34.3)	1.25 (0.64, 2.45), p = 0.577
**Smoking status**			
Current smoker	23/41 (56.1)	25/52 (48.1)	1.17 (0.79, 1.73), p = 0.532
Ex-smoker	49/86 (57.0)	35/86 (40.7)	1.40 (1.02, 1.92), p = 0.047
Non-smoker	30/58 (51.7)	31/66 (47.0)	1.10 (0.77, 1.57), p = 0.719
Missing	0/1	1/1 (100.0)	

mITT modified intent to treat: *LTCF*, *long-term care facility; ICU intensive care unit;* APACHE, Acute Physiology and Chronic Health Evaluation; ACE, angiotensin-converting-enzyme inhibitor; ARB, angiotensin receptor blocker; *NSAID*, nonsteroidal anti-inflammatory drug; *MRSA*, *methicillin-resistant Staphylococcus aureus; MDRD* Modification of Diet in Renal Disease; CI, confidence interval;

The unadjusted and adjusted odds ratios for clinical success at EOS for the factors that remained following the correlation analysis are shown in Table 3. Predictors of clinical success in the multivariate model included absence of vasopressors (OR 2.30, 95% CI: 1.303, 4.069), unilateral involvement by chest x-ray (OR 1.70, 95% CI: 1.078, 2.681), normal renal function (eGFR 30–80 vs >80 OR 0.48, 95% CI: 0.303, 0.750), and treatment with linezolid. (Table 3) Specifically, the OR for clinical success with linezolid in the adjusted analyses measured 1.55 (95% CI: 1.013, 2.355).

**Table 3:** Unadjusted and adjusted odds ratios of baseline parameters and treatment regimen on clinical success at EOS.

Variables	Clinical Success Unadjusted OR (95% CI)	Clinical Success Adjusted OR (95% CI)^1^ from multivariate regression model
Vasopressors (no vs yes)	2.45 (1.411, 4.252)	2.30 (1.303, 4.069)
Chest X-ray (unilateral vs. bilateral)	1.68 (1.089, 2.585)	1.70 (1.078, 2.681)
Treatment (LZD vs VAN)	1.49 (1.001, 2.223)	1.55 (1.013, 2.355)
eGFR, mL/min/1.73m^2^ (<30 vs >80)	0.73 (0.354, 1.511)	0.85 (0.404, 1.797)
eGFR, mL/min/1.73m^2^ (30–80 vs >80)	0.44 (0.286, 0.691)	0.48 (0.303, 0.750)
Pleural Effusion (no vs yes)	0.79 (0.530, 1.179)	
Cardiac comorbidity (no vs yes)	1.71 (1.139, 2.571)	
Pathogen other than MRSA (no vs yes)	1.38 (0.927, 2.064)	
Race (Asians vs Other)	2.35 (0.738, 7.474)	
Race (Black vs Other)	1.69 (0.524, 5.438)	
Race (White vs Other)	1.33 (0.460, 3.841)	
Gender (female vs male)	1.15 (0.756, 1.736)	
Oncology comorbidity (no vs yes)	1.62 (0.834, 3.133)	
Bacteremia–blood (no vs yes)	1.78 (0.995, 3.185)	
Smoking Status (current smoker vs. non-smoker)	1.12 (0.675, 1.851)	
Smoking status (ex-smoker vs. non-smoker)	1.01 (0.639, 1.610)	
Hepatobiliary comorbidity (no vs yes)	1.21 (0.687, 2.120)	
Diabetes comorbidity (no vs yes)	1.18 (0.788, 1.773)	
Type of Patient (medical vs. trauma)	0.92 (0.549, 1.538)	
Type of Patient (surgical vs. trauma)	0.83 (0.459, 1.516)	
Baseline Diuretics (no vs yes)	1.03 (0.663, 1.600)	
Age (per 1 year increment)	1.00 (0.985, 1.007)	
Weight (per 1 kilogram increment)	1.00 (0.994, 1.012)	
Renal / urinary comorbidity (no vs yes)	1.13 (0.755, 1.702)	
Pulmonary comorbidity (no vs yes)	1.18 (0.771, 1.795)	
Apache II score (baseline) (per 1 unit increase)	0.97 (0.934, 0.998)	
Patient in the ICU at Baseline (no vs yes)	1.21 (0.680, 2.150)	
Corticosteroids at baseline (no vs yes)	1.07 (0.665, 1.726)	
Gastrointestinal comorbidity (no vs yes)	0.91 (0.609, 1.353)	
Baseline Anaerobic Coverage (no vs yes)	0.84 (0.560, 1.246)	
Baseline ACE/ARBs (no vs yes)	0.58 (0.324, 1.047)	
LTCF Resident at baseline (no vs yes)	1.10 (0.437, 2.767)	

^1^ Hosmer-Lemeshow test p = 0.17

CI, confidence interval; eGFR, estimated glomerular filtration rate calculated by the Modification of Diet in Renal Disease equation; EOS, end of study; EOT, end of treatment; MRSA, methicillin-resistant Staphylococcus aureus; OR, odds ratio.

## Discussion

In this analysis, a higher clinical success rate was observed for patients treated with linezolid compared to those treated with vancomycin. Clinical success rates in the stratified analysis favored linezolid for the majority of baseline characteristics and were significantly different among a few strata. These results were consistent with the multivariate analysis. Treatment with linezolid compared to vancomycin for MRSA pneumonia was associated with higher rates of clinical success even after adjustment for other potential explanatory factors at baseline. In addition, patients not receiving vasopressors, only having unilateral involvement compared to multilobe involvement, and patients with normal renal function compared to abnormal renal function were more likely to be classified as clinical successes.

The rate of clinical success was not altered whether or not the patient had bacteremia or mechanical ventilation. In addition, these factors were not a significant predictor of clinical success. Some have suggested that imbalance in the rates of bacteremia and mechanical ventilation may account for the significant treatment difference seen in the primary analysis of this trial comparing linezolid to vancomycin.[8,9] The present secondary analysis, however, suggests this is not the case and demonstrates that linezolid remains associated with greater cure rates even when adjusted for these factors.

Patients were only included in this secondary analysis if they received the study treatment, had a culture confirming MRSA, and a documented result at EOS. This population differs slightly from both the modified intent to treat population and per protocol population in the original trial. We chose to use these criteria in order to be more inclusive of the patients that were exposed to the treatment and in order to ensure the most complete final follow up outcome.

This secondary analysis has several limitations. First, since this is a retrospective evaluation it can only be considered exploratory. The experimental design in the original trial was not specifically developed to evaluate individual factors and may not be ideal for assessing the importance of some baseline characteristics due to low prevalence of these various conditions. In other words, we may have lacked power to assess the significance of several potential co-variates. However, a regression model is a valid analytic approach for addressing these questions using the existing data set.

Second, these multivariate analyses infer based on associations and cannot establish a causal relationship between individual identified predictors and clinical success. Similarly, the potential predictors were evaluated for the period of hospitalization only. We also acknowledge that not all patients in the modified intent to treat population had a clinical response available at EOS and so these patients had to be excluded from our analyses, which may have diminished the benefits of randomization that exists within the entire modified intent to treat population. Finally, as this was a retrospective analysis of a clinical trial, the results should be interpreted with caution, and prospective studies are needed to validate the preliminary trends suggested in this analysis.

From a clinical standpoint, identifying reliable predictors of outcome and who might benefit more from one therapy versus another can help inform treatment decisions. The interaction between the pathogen, disease course, and treatment response that occurs in MRSA pneumonia patients is complex. Our findings provide additional data on the association between treatment and outcome after adjusting for known factors associated with MRSA pneumonia.
